# Energy Drinks and the Risk of Cardiovascular Disease: A Review of Current Literature

**DOI:** 10.7759/cureus.1322

**Published:** 2017-06-07

**Authors:** Muhammad A Mangi, Hiba Rehman, Muhammad Rafique, Michael Illovsky

**Affiliations:** 1 GME Internal Medicine, Orange Park Medical Center; 2 Anesthesiology, Liaquat National Hospital; 3 Cardiology, Orange Park Medical Center

**Keywords:** energy drink, cardiovascular diseases, cardiac arrhythmias

## Abstract

Energy drinks (EDs) are commonly used as a dietary supplement by young adolescents and adults. They are often used as a source of energy in order to enhance physical and mental performance. EDs contain a variety of substances, but caffeine is the main component. Safety has been the biggest concern associated with consuming EDs. Case reports, observational studies, and meta-analyses have been done in order to determine the effects of EDs on cardiovascular changes. The detrimental effects of EDs are cardiac arrhythmias, myocardial infarction, prolonged QT interval, aortic dissection, and death. In this article, we review case reports, observational studies, and meta-analyses of EDs and the risk of cardiovascular events and mortality. We also review active ingredients, pharmacokinetics, and the mechanism of action of EDs.

## Introduction and background

Energy drinks (EDs) are more commonly used by the young population as a source of dietary supplement. EDs containing caffeine and other stimulants were introduced in the United States of America (USA) in 1997. They are consumed by 30-50% of the young population of the USA [1]. The popular brands in the USA are Red Bull, Rockstar, Monster, Full Throttle and Amp. These are often used to improve weight loss, athletic performance, energy level, concentration, and decrease the aftereffects of alcohol [2-3]. The common ingredients of EDs are caffeine, sugar, taurine, vitamin B complex, guarana, and ginseng. Most of these ingredients are not well studied. An energy shot is the concentrated form of an ED and is often sold in a smaller bottle. Instead of being labeled as beverages, these substances are labeled as dietary supplements. Under the Dietary Supplement Health and Education Act of 1994, dietary supplement ingredients do not require United States Food and Drug Administration (FDA) approval. Subsequently, the FDA has a limited a role in the regulation of EDs.

The safety of EDs and shots remains debatable, as there are a number of cases where they have produced adverse events in patients. Recently, ED-associated emergency visits have sharply increased [3]. They are often found in combination with other substances, such as alcohol and drugs of abuse, which further potentiates the effect of EDs [4]. EDs have been recognized to cause cardiovascular changes, particularly cardiac arrhythmias [1]. These cardiovascular changes can lead to morbidity and mortality [5]. This article will focus on cardiovascular changes associated with EDs, ingredients of EDs, pharmacokinetics, and the mechanism of EDs.

## Review

### Ingredients of EDs

Caffeine is one of the main stimulants in EDs. The concentration varies in different products [6-7]. Other components like guarana, taurine, theophylline, ginkgo biloba, ginseng, vitamins, L-carnitine etc. are also present in variable amounts. These ingredients also increase one's energy and stimulate mental performance [7-8]. Guarana is a caffeine-containing plant which contains higher concentrations of caffeine. This is one of the additional sources of caffeine in EDs [9].

### Pharmacokinetics of EDs

Caffeine is completely and rapidly absorbed orally with an approximately 4.5 hours half-life [10]. After absorption, caffeine undergoes metabolism in the liver into methyl-xanthine products [11-12]. The caffeine follows zero order kinetics after converting into methyl-xanthine [13]. Hence the rate of elimination is constant, not affected by drug intake and plasma concentration of the drug. This results in a longer recovery time after a caffeine overdose. Caffeine metabolism is affected by other substances like chronic alcoholism, which decreases the metabolism and hence increases the half-life of caffeine. Nicotine increases the metabolism of caffeine and hence decreases the half-life [14-15].

### Mechanism of EDs

Caffeine in the EDs is metabolized into methyl-xanthine, which acts by inhibiting phosphodiesterase, antagonize adenosine receptor and increases catecholamine secretion. At therapeutic doses, the physiologic effects include tachycardia, increased blood pressure, diuresis, vomiting, diarrhea, bronchodilation, gastric acid secretion and central nervous system stimulation [16]. In an overdose condition, tachycardia followed by arrhythmias and hypotension can occur. The main toxicity caused by caffeine is due to the increase in intracellular calcium concentration, which leads to a catecholamine surge that sensitizes dopamine receptors resulting in supraventricular or ventricular arrhythmias and death [17-19]. Caffeine primarily affects vasculature while other ingredients in the EDs primarily affect the heart to cause cardiovascular changes [20]. The effect on the heart can be detrimental. Steinke, et al. reported the increase in heart rate and blood pressure after few hours of ingestion of EDs [21]. Similarly, Grasser, et al. reported that Red Bull was associated with increased heart rate and cardiac output [22]. Baum, et al. demonstrated the effects of Red Bull on the heart via echocardiography [23]. In this study, Baum, et al. compared Red Bull-induced echocardiographic changes before and after exercise. Interestingly, they found that Red Bull increased stroke volume significantly before and after exercise compared to individuals in the control group. Similarly, Menci, et al. found increased ventricular contractility after ED consumption [24]. Based on the above studies, we can summarize that EDs cause increased myocardial contractility, heart rate, and stroke volume, while caffeine causes increased peripheral vascular resistance [20]. The other components of EDs also contribute to cardiovascular changes. The sugar component of EDs causes dyslipidemia, weight gain, insulin resistance and pro-inflammatory activity [25].

### EDs and risk of cardiovascular events

The cardiovascular risk associated with ED is debatable. Case reports have shown that EDs causes arrhythmias, but the mechanism is not well known. Some studies favor that EDs do not cause arrhythmias [26-27], but some cases reports have reported arrhythmias only at higher doses [1, 28]. It is unknown what makes some people more susceptible to arrhythmias. It may be that some individuals are genetically susceptible to the effects of EDs, resulting in arrhythmias or it may be the actual acute ingestion of EDs putting the patient at risk of arrhythmias. There is also a lack of studies regarding chronic ingestion of ED and the long-term risk of arrhythmias. There are a variety of cardiovascular changes seen in patients taking EDs, with the most common being arrhythmias. Other cardiovascular changes (Table 1) associated with EDs are coronary vasospasm [29], ST elevation [30], prolonged QT interval [31], aortic aneurysm dissection [32], cardiac arrest [33], cardiomyopathy [34], and acute coronary thrombosis [35].

**Table 1:** Cardiovascular changes with energy drink

Cardiovascular diagnosis
Supraventricular arrhythmias (​*Atrial fibrillation, Narrow complex tachycardia) *
Ventricular arrhythmias (Ventricular tachycardia, Ventricualr fibrillation, Torsade de pointes) * *
Prolonged QT interval
Myocardial ischemia and infarction
Coronary vasospasm
Coronary thrombosis
Cardiomyopathy
Aortic Dissection

EDs and Risk of Supraventricular Arrhythmias

There have been case reports linking EDs to the risk of atrial fibrillation, but there is an unclear association between EDs and the risk of atrial fibrillation [1]. Some studies have shown that caffeine stimulates cardiac adenosine, which inhibits cardiac fibroblast production in vitro, and hence inhibits remodeling after myocardial infarction [36-37], which can be a contributing factor for the development of atrial fibrillation. However, there are observational studies and meta-analyses that demonstrate that caffeine protects from atrial fibrillation [38] (Table 2). Garcia, et al. demonstrated caffeine as an antioxidant [39], which may act as a protective factor in preventing atrial fibrillation. There is no data for chronic ingestion of ED and risk of atrial fibrillation.

Nagajothi, et al. reported a case of a 23-year-old man with chest tightness after consuming EDs. On arrival, the initial electrocardiogram (EKG) showed narrow complex tachycardia with a ventricular rate of 219 beats/minute. Carotid sinus massage and Valsalva maneuvre were tried to terminate the tachycardia but without success. Later on, adenosine was administered and the patient converted into normal sinus rhythm [40].

EDs and Risk of Prolonged QT Interval

There are a couple of cases that report caffeine ingestion posing a risk of prolonging the QT interval. Other ED components like ginseng have also been reported to cause QT prolongation in a young patient, which progressed to torsades de pointes [32]. Likewise, Redline and SPIKE Shooter which contain yohimbine can also prolong the QT interval and leads to the risk of arrhythmias [41-43]. The proposed mechanism is that yohimbine leads to increased adrenergic stimulation [41-43]. Yohimbine inhibits presynaptic alpha 2 receptors, hence leading to an increased central adrenergic surge. This adrenergic surge further leads to a vasopressor response, tachycardia, and QT prolongation. Other ingredients like ephedra have been seen to cause multiple cardiovascular problems, hence it was removed from the market [44]. The consequence of QT prolongation is torsades de pointes, which can further lead to ventricular fibrillation and cardiac arrest.

EDs and Risk of Ventricular Arrhythmias

There are case reports of ventricular arrhythmias and sudden cardiac death secondary to EDs (Table 2). These were reported in both patients with and without structural heart disease. Goldfarb, et al. described a case of a young man who was found by emergency medical services in ventricular fibrillation and regained a rhythm after defibrillator shock [45]. On questioning, it was found that the patient smokes marijuana and consumed caffeine-rich EDs. Subsequent cardiac work was unremarkable including echocardiography, cardiac magnetic resonance imaging, coronary angiography and an electrophysiological study. Cannon, et al. described a case of a young woman with the history of mitral valve prolapse who developed ventricular fibrillation and died after consuming EDs containing guarana, ginseng, and caffeine [19]. Later on, this drink was removed from the market due to safety concerns. Rottlaender, et al. described a case of a young woman who developed cardiac arrest within four hours after ingestion of an ED [34]. The patient developed QT prolongation and subsequently went into torsades de pointes and ventricular fibrillation. Genetic testing confirmed that the patient had type 1 long QT syndrome. Dufendach, et al. described an adolescent woman who consumed EDs every other day for two weeks before coming to the emergency department with chest pain and palpitation. She was found to have a prolonged QT interval, and subsequent genetic testing confirmed long QT syndrome [46]. Similarly, Rutledge, et al. described a case of a young adult male who developed ventricular fibrillation after drinking EDs along with alcohol [47]. Post-resuscitation EKG showed Brugada type 1 pattern. Cases of ventricular arrhythmias have been reported in a patient with structural heart disease, like ventricular tachycardia in a patient with tetralogy of Fallot after consuming EDs [48].

**Table 2:** Cardiac arrhythmia with energy drink

Age (years)	Gender	Race	Author	Year Published	Study design	Finding
Median 53	Female	Majority Caucasian	Conen, et al. [26]	2010	Prospective cohort study	Not associated with increased risk of atrial fibrillation
Median Male/Female 62.0±9.8/ 62.8±10.4	Male/Female 4231/5409	Not described	Shen, et al. [27]	2011	Prospective cohort study	Not associated with increased risk of atrial fibrillation
14 16	Male	Caucasian	Di Rocco, et al. [28]	2011	Two case reports	Increased risk of atrial fibrillation
23	Female	Not described	Nagajothi, et al. [40]	2008	Case report	Increased risk of narrow complex tachycardia
43	Female	Not described	Torbey, et al. [31]	2011	Case report	Increased risk of prolonged QT interval
-	Animal	Animal	Arnar, et al. [43]	2007	Animal study	Increased risk of ventricular tachycardia
25	Female	Not described	Cannon, et al. [19]	2001	Case report	Increased risk of ventricular fibrillation
22	Female	Not described	Rottlaender, et al. [33]	2012	Case report	Torsades pointes, ventricular fibrillation
24	Male	Not described	Rutledge, et al. [47]	2012	Case report	Ventricular fibrillation in patient with history of Brugada syndrome
45	Male	Not described	Ward, et al. [48]	2014	Case report	Ventricular fibrillation
-	-	-	Goldfarb, et al. [45]	2014	Systemic review	Increased risk of atrial fibrillation, supraventricular arrhythmia, prolonged QT interval, torsades pointes, ventricular tachycardia, ventricular fibrillation, ST elevation

EDs and Risk of Myocardial Ischemia or Infarction

Cases have been reported where EDs have been found to cause myocardial ischemia and infarction (Table 3). Berger, et al. described a case of a young man who developed a cardiac arrest seven hours after drinking seven to eight cans of EDs [30]. The sinus rhythm was restored after two defibrillator shocks, 1 mg of epinephrine and 1 mg of atropine. On arrival to the hospital, the initial EKG showed a normal sinus rhythm with anteroseptal ST elevation and reciprocal inferior ST depression. Israelit, et al. describes a case of a young male who was brought to the hospital from a night club with crushing chest pain after drinking 20 cans of EDs [31]. On arrival to the hospital, the EKG showed widespread ST segment elevation. While waiting for percutaneous coronary intervention (PCI), the patient developed ventricular fibrillation and died. The possible mechanism leading to myocardial infarction include endothelial dysfunction and platelet aggregation [49].

EDs and Risk of Cardiomyopathy

To the best of the authors’ knowledge, there is one case report of EDs causing a cardiomyopathy [35]. This was the case of a 24-year-old young male who presented to the emergency department with chest pain and palpitation after consuming EDs. The patient had sinus tachycardia along with frequent runs of supraventricular tachycardia and ventricular tachycardia. A chest X-ray showed pulmonary edema, and an echocardiogram demonstrated an ejection fraction (EF) of 35%. This energy drink contained sympathomimetic substances, specifically caffeine and 1,3-dimethylamylamine (DMAA). Caffeine has been documented to cause arrhythmias by acting at adenosine receptors and inducing a catecholamine surge, while DMAA is structurally similar to amphetamine. In vessels, amphetamines have norepinephrine activity while in the brain they have stimulant activity.

EDs and Risk of Aortic Dissection

Jonjev, et al. described the detrimental side effect of ED consumption [32]. The authors reported three case reports of aortic dissection in high-risk cardiovascular patients who developed aortic dissection after drinking EDs. All of these cases required surgical correction. In the first case, a 54-year-old Caucasian male with a history of obesity and uncontrolled hypertension was admitted with chest pain and shortness of breath. The patient further described consuming four to five EDs per night. On arrival, his blood pressure was 190/110 mm Hg and his heart rate was 110 beats per minute. In the second case, a 26-year-old Caucasian male with a history of bicuspid aortic valve and a 5 cm ascending aortic aneurysm was admitted with chest pain. The patient described that the chest pain started after he drank five to six EDs. The third case was a 48-year-old Caucasian male with a family history of hypertension and myocardial infarction who was admitted with chest pain. The patient reported that the chest pain started after drinking several EDs. The possible mechanism for EDs provoking aortic dissection is due to caffeine-induced high blood pressure. Further studies are lacking in regard to ED association with aortic dissection.

**Table 3:** Other cardiovascular changes with energy drink

Age (years)	Gender	Race	Author	Year Published	Study design	Finding
28	Male	Not described	Berger, et al. [29]	2009	Case report	Anteroseptal ST segment elevation with reciprocal inferior ST segment depression, ventricular fibrillation
24	Male	Caucasian	Israelit, et al. [30]	2012	Case report	Widespread ST segment elevation
24	Male	Not described	Kaoukis, et al. [34]	2012	Case report	Hypokinesis of all basal left ventricular segments with apical sparing and an ejection fraction of 35%
54, 26, 48	Male	Caucasian	Jonjev, et al. [32]	2013	Case series	Aortic dissection

## Conclusions

The previously mentioned studies suggest the association between EDs and cardiovascular changes including cardiac arrhythmias, prolonged QT interval, ventricular arrhythmias, cardiac arrest, cardiomyopathy, myocardial ischemia, infarction, aortic dissection, and death. Some of these case reports were reported on patients who had underlying structural heart disease, inherited cardiac problems, and abuse of other substances along with EDs. Based on case reports, limited observational studies, and the meta-analysis, it is suggested EDs can be dangerous when used alone or in combination with other substances of abuse. Well-designed prospective studies are required to definitely prove the association of EDs with cardiovascular events.
